# CCDC65 Mutation Causes Primary Ciliary Dyskinesia with Normal Ultrastructure and Hyperkinetic Cilia

**DOI:** 10.1371/journal.pone.0072299

**Published:** 2013-08-26

**Authors:** Amjad Horani, Steven L. Brody, Thomas W. Ferkol, David Shoseyov, Mollie G. Wasserman, Asaf Ta-shma, Kate S. Wilson, Philip V. Bayly, Israel Amirav, Malena Cohen-Cymberknoh, Susan K. Dutcher, Orly Elpeleg, Eitan Kerem

**Affiliations:** 1 Department of Pediatrics, Washington University School of Medicine, St. Louis, Missouri, United States of America; 2 Department of Medicine, Washington University School of Medicine, St. Louis, Missouri, United States of America; 3 Department of Cell Biology and Physiology, Washington University School of Medicine, St. Louis, Missouri, United States of America; 4 Department of Pediatrics, Hadassah Hebrew University Medical Center, Jerusalem, Israel; 5 Monique and Jacques Roboh Department of Genetic Research, Hadassah Hebrew University Medical Center, Jerusalem, Israel; 6 Department of Mechanical Engineering and Material Science, Washington University, St. Louis, Missouri, United States of America; 7 Department of Pediatrics, Ziv Medical Center, Bar-Ilan University, Safed, Israel; 8 Department of Genetics, Washington University School of Medicine, St. Louis, Missouri, United States of America; University of Tübingen, Germany

## Abstract

**Background:**

Primary ciliary dyskinesia (PCD) is a genetic disorder characterized by impaired ciliary function, leading to chronic sinopulmonary disease. The genetic causes of PCD are still evolving, while the diagnosis is often dependent on finding a ciliary ultrastructural abnormality and immotile cilia. Here we report a novel gene associated with PCD but without ciliary ultrastructural abnormalities evident by transmission electron microscopy, but with dyskinetic cilia beating.

**Methods:**

Genetic linkage analysis was performed in a family with a PCD subject. Gene expression was studied in *Chlamydomonas reinhardtii* and human airway epithelial cells, using RNA assays and immunostaining. The phenotypic effects of candidate gene mutations were determined in primary culture human tracheobronchial epithelial cells transduced with gene targeted shRNA sequences. Video-microscopy was used to evaluate cilia motion.

**Results:**

A single novel mutation in *CCDC65*, which created a termination codon at position 293, was identified in a subject with typical clinical features of PCD. CCDC65, an orthologue of the *Chlamydomonas* nexin-dynein regulatory complex protein DRC2, was localized to the cilia of normal nasal epithelial cells but was absent in those from the proband. *CCDC65* expression was up-regulated during ciliogenesis in cultured airway epithelial cells, as was DRC2 in *C. reinhardtii* following deflagellation. Nasal epithelial cells from the affected individual and *CCDC65*-specific shRNA transduced normal airway epithelial cells had stiff and dyskinetic cilia beating patterns compared to control cells. Moreover, Gas8, a nexin-dynein regulatory complex component previously identified to associate with CCDC65, was absent in airway cells from the PCD subject and *CCDC65*-silenced cells.

**Conclusion:**

Mutation in CCDC65, a nexin-dynein regulatory complex member, resulted in a frameshift mutation and PCD. The affected individual had altered cilia beating patterns, and no detectable ultrastructural defects of the ciliary axoneme, emphasizing the role of the nexin-dynein regulatory complex and the limitations of certain methods for PCD diagnosis.

## Introduction

Primary ciliary dyskinesia (PCD) (OMIM# 244400) is a genetic disorder characterized by impaired ciliary function, leading to diverse clinical manifestations that include chronic sinopulmonary disease, persistent middle ear effusions, laterality defects, and infertility. Current diagnostic studies include transmission electron microscopy (EM), cilia motion analysis, nasal nitric oxide (NO) measurements, and more recently, genetic testing for disease-causing mutant alleles. Most of these techniques are only available at specialized centers [1], [2]. Until recently, transmission EM has been considered the cornerstone for PCD diagnosis based on the initial descriptions of structural defects in ciliary axonemes from affected subjects [3]. However, there is a current consensus that the reliance on EM alone is inadequate to diagnose PCD [4].

The complex genetics of PCD is rapidly unfolding so that the identification of PCD-causing mutations could lead to the development of comprehensive genetic testing to overcome current diagnostic limitations. In most cases, the disease is expressed in an autosomal recessive pattern, and theoretically, mutations in any of nearly 2000 proteins that assemble or constitute a motile cilium could potentially cause disease [5]. To date, 19 genes have been associated with PCD [6]–[28]. These genes can be classified into three groups: genes encoding proteins that are components of the outer or inner dynein arms including *DNAH5*, *DNAI1*, *DNAL1*, *DNAI2*, *TXNDC3* and *DNAH11*; genes encoding proteins implicated in axonemal organization of the central pair microtubules, radial spokes and nexin-dynein regulatory complex (N-DRC) including *CCDC39*, *CCDC40*, *CCDC164*, *CCDC103*, *CCDC114, RSPH9*, *RSPH4A*, and *HYDIN*; and genes encoding proteins that are cytoplasmic and do not localize to the ciliary axoneme, including *HEATR2*, *DNAAF1*, *DNAAF2*, *DNAAF3* and *LRRC6* [11], [12], [18], [21], [23]–[25], [27]. The cytoplasmic proteins are presumed to have roles in cilia assembly or protein transport, and mutations lead to ultrastructural abnormalities. Of note, subjects with *DNAH11* (MIM 603339) mutations present with a clinical phenotype consistent with PCD, but with no ultrastructural defects detectable using standard transmission EM [15]. Mutations in other genes such as *CCDC39* (MIM 613798) and CCDC40 (MIM 613799) produce inconsistent ultrastructural abnormalities characterized by disordered microtubules in only some respiratory cells [14], [19], [20], which underscores the limitations of EM as a diagnostic test for PCD [15].

Another diagnostic approach utilized for PCD has been the detection of immotile or paralyzed cilia. However, it is increasingly recognized that this is not a consistent finding for all cases of PCD since a slow or altered pattern of ciliary motility may instead be present [13], [28]. To detect these changes, specialized instrumentation and specific expertise is required. Furthermore, secondary effects on cilia beat related to the presence of infection, inflammation, or smoke exposure may obscure the findings [29].

Here, we report an individual with PCD with normal ciliary ultrastructure as determined by electron microscopy, and altered ciliary beating. Linkage analysis on the proband and his parents revealed a single homozygous frameshift mutation in *CCDC65*, also known as DRC2, which is a component of the nexin-dynein regulatory complex (N-DRC). The latter is proposed to regulate the activity of the dynein motors [30], [31]. Mutations in DRC1, another N-DRC protein, were previously associated with PCD secondary to altered cilia beat pattern [28].

## Materials and Methods

### Subjects and Genetic Analysis

**Figure 1:**
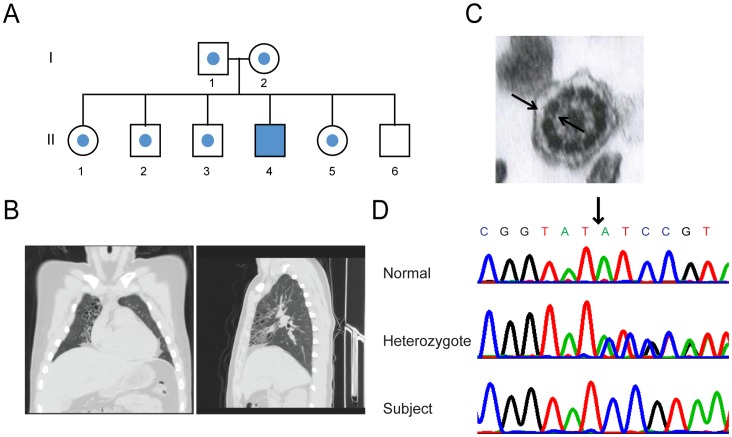
Family pedigree and genetic analyses. (**A**) Pedigree of subject with PCD from an Ashkenazi-Jewish family. The closed symbol represents the affected individual and symbols with central closed circles represent heterozygous individuals. (**B**) Computerized x-ray tomography of the chest of the affected individual showing bronchiectasis. (**C**) Transmission electron microscopy of cilia from an affected individual failed to show structural defects. (**D**) Chromatogram showing the nucleotide sequence of the *CCDC65* exon 6 adjacent to the mutation site, at position c.876_877delAT (ch12:47598804-5), which is predicted to result in frameshift leading to a premature termination codon.

A subject with clinical features consistent with PCD, from a non-consanguineous family of Ashkenazi-Jewish descent was studied (**Figure 1A and 1B**). Genetic linkage analysis was performed on the affected individual, siblings, and parents (**Figure 1A**). Because of the common ethnic origin of the parents in this family, we assumed a founder mutation transmitted in an autosomal recessive manner. A search for homozygous regions was performed on the DNA sample from the proband using the GeneChip Human Mapping 250K Nsp Affymetrix Array as previously described [**32**].

### Ethics Statement

All individuals or their parents provided written informed consent for diagnostic evaluation and genetic characterization. The Hadassah-Hebrew University Human Subjects Committee and Washington University Human Research Protection Office approved the study protocol. Also used in this study were anonymized human airway epithelial cells from surgical excess of large airways of lung donated for transplantation at Washington University in St. Louis that were trimmed during the transplant procedure. Research using cells originating from deidentified cadaver specimens (surgical excess of large airways of lung) are exempt from regulation and is not governed by NIH regulation 45 CFR Part 46.

### Clinical Assessment

Disease severity assessment was quantified by chest computerized x-ray tomography (CT) scans using the Brody score for grading bronchiectasis, adapted from its original use in cystic fibrosis bronchiectasis to non-CF bronchiectasis, including PCD [33]. Nasal nitric oxide was measured using a CLD 88sp NO analyzer (ECO MEDICS AG, Switzerland) according to the manufacturer protocols and as recommended by the joint American Thoracic Society and European Respiratory Society consensus statement [34].

### Airway Epithelial Cells

Nasal epithelial cells from subjects were obtained from the inferior turbinate by cytology brush [35]. Human tracheal airway epithelial cells (hTEC) were isolated from surgical excess of large airways (tracheobronchial segments) of normal lungs donated for transplantation. Cells were expanded in culture then seeded on supported filters (Transwell, Corning Inc., Corning, NY) and re-differentiated using air-liquid interface conditions [36]. Cell preparations were maintained in culture for four to ten weeks.

### Video-microscopy Ciliary Motion Analysis

Nasal epithelial cells were collected from subjects with PCD by nasal brushing and examined using previously published protocols [4]. Samples were suspended in cell culture media in a 24-well plate, and then imaged in laboratories at the clinical site using an inverted microscope equipped with a phase contrast objective (Eclipse TS-100; Nikon Corp. Tokyo, Japan) at 400× magnification. Ten video sequences of each sample were recorded at room temperature within 10 minutes of sample collection. Images were captured at 120 frames per seconds, each for 3 seconds in length, using a high-speed video camera (scA640, Basler AG, Ahrensburg, Germany). Images were processed with the Sisson-Ammons Video Analysis system (Ammons Engineering, Mt. Morris, MI, USA) using established methodologies [37]. Ciliary beating patterns of the PCD subject were qualitatively evaluated using slow motion playback (at 1/8^th^ speed) of video sequences.

Video-microscopy of cultured airway epithelial cells was performed at Washington University. Cultured airway epithelial cells grown at an air-liquid interface were first scraped from Transwell filters. Video-microscopy of ciliated epithelial cells was performed using an inverted microscope with a phase contrast objective (Eclipse Ti-U; Nikon, Melville, NY) enclosed in a customized environmental chamber maintained at 37°C to minimize secondary effects on cilia activity from temperature fluctuations. The motion of cilia on cells clusters was viewed from the side and captured by a high-speed video-microscopy. Cilia velocity fields within groups of cilia were evaluated by estimating optical flow in a region of interest containing the cilia [38], [39]. The Fast Fourier Transform (FFT) was then used to find a velocity spectrum, which describes velocity amplitude as a function of frequency. CBF was estimated as the frequency corresponding to the highest peak of the velocity spectrum. We believe that estimates of CBF from velocity fields are more reliable than estimates from image intensity fields, since velocities of forward and recovery strokes differed more clearly than intensities.

### Gene Silencing of Airway Epithelial Cells

*CCDC65* expression was silenced using an RNAi approach in primary airway epithelial cells [11], [40]. Human airway epithelial cells were transduced with non-targeted or *CCDC65*-specific shRNA sequences using a recombinant lentivirus vector that contained a cassette to confer puromycin resistance. The shRNA-targeted sequences used were those generated by The RNA Consortium shRNA library in the pLKO.1 plasmid [40] provided by the shRNA core facility of the Children’s Discovery Institute of St. Louis Children’s Hospital and Washington University School of Medicine. Vesicular stomatitis virus envelope glycoprotein (VSV-G)-pseudotyped virus was generated by three-plasmid cotransfection of HEK 293T cells using Fugene 6 (Roche, WI). The virus generated in airway cell medium was filtered and used to transduce hTEC. Tranduced cells were then selected by adding puromycin (2.5 µg/ml) to the culture medium for 5 days [41]. Once confluent, airway epithelial cells were grown at an air-liquid interface. shRNA sequences used were: CCAAGGAGTTTGAGACAGAAA (shRNA#1); CCAAACATTTGAACGAGTGGT (shRNA#2); GCAAGATATCTTCATGGCCAT (shRNA#3); and GCTGCTTCTGTTTCAGCAGAA (shRNA#4). A non-targeted sequence subcloned into a plasmid containing the yellow fluorescent protein (YFP) reporter and the puromycin resistance genes (gift from Y. Feng and G.D. Longmore, Washington University, St. Louis, MO) was used as a control sequence [42].

### Reverse Transcriptase-polymerase Chain Reaction Assay

RNA was isolated from cells using the Illustra RNAspin kit (GE Healthcare, Buckinghamshire, UK), reverse transcribed using a cDNA Reverse Transcription Kit, then amplified and quantified using the TaqMan Fast Universal PCR Master Mix (both from Applied Biosystems, Carlsbad, CA) with SYBR green in a Lightcycler 480 (Roche, Indianapolis, IN) [43]. Target gene expression was normalized to Ornithine decarboxylase antizyme (*OAZ1*) expression. *CCDC65* and *Foxj1* expression were assessed using the following oligonucleotide primer sets: human *CCDC65*, 5′-TCCTGTTCGAGGGCTGAGAT and 5′-GGGGATTGGATCCGGGAAAG; *FOXJ1*, 5′-CCCGACGACGTGGACTAC and 5′-GGCGGAAGTAGCAGAAGTTG.

### Epithelial Cell Immunofluorescent Staining and Immunoblot Analyses

Human tracheobronchial epithelial cells from non-PCD subjects differentiated at an air-liquid interface [36] or nasal airway epithelial cells obtained from the inferior nasal turbinate were fixed and immunostained as previously reported [36], [44]. Primary antibodies and dilutions used included rabbit anti-CCDC65 (1∶100, Novus Biologicals, Littleton, CO), acetylated α-tubulin (1∶5000, clone 6-11-B1, Sigma Aldrich), GAS8 (1∶100, SAB1101111, Sigma Aldrich), and were detected with secondary antibodies conjugated to Alexa Fluor dyes (A-21202, A-21206, A-31570 and A-31572; Life Technologies, Grand Island, NY). Nuclei were stained using 4′, 6-diamidino-2-phenylindole (DAPI) 1.5 µg/mL (Vector laboratories, Burlingame, CA). Cells were imaged using a Leica DM5000 epifluorescent microscope with band-pass filter cubes optimized for the secondary dyes (Wetzlar, Germany) and equipped with a Retiga 200R CCD camera (Q-Imaging) interfaced with Q-Capture Pro software (Q-Imaging). Images were adjusted globally using Photoshop (Adobe Systems, San Jose, CA).

For immunoblot analyses, lysed cells were resolved by SDS-PAGE (7.5%) then transferred to PVDF membranes. The blots were incubated in blocking buffer of 1X TBS, 0.1% Tween-20 with 5% w/v nonfat dry milk, followed by incubation in blocking buffer with the CCDC65 antibody (1∶100 dilution). Signal was detected using enhanced chemiluminescence.

### *Chlamydomonas* Flagellar Deflagellation

Cells were grown on low-sulfur medium for 3 days in light and 2 days in the dark, and then differentiated into gametes over 4 hours with the use of medium that lacks nitrogen; deflagellation was induced by a pH shock [45]. Expression of *CCDC65* was assessed following deflagellation at several time points and compared to pretreatment values as described [46].

### Statistical Analyses

Data are expressed as mean ± standard deviation (SD). Statistical comparisons between groups were made using unpaired two-tailed Student’s *t* tests or single factor analysis of variance (ANOVA) with Bonferroni correction.

## Results and Discussion

The proband was an 18-year old male of Ashkenazi-Jewish descent who presented with recurrent upper and lower respiratory tract infections, beginning in early infancy. His parents and five siblings were unaffected (**Figure 1A**). Pulmonary function measures were normal, but CT of the chest revealed diffuse cystic bronchiectasis, primarily involving the right middle lobe, lingula, and both lower lobes (total Brody score, 27.75 [33]) (**Figure 1B**). Imaging of the paranasal sinuses demonstrated opacification of the maxillary and ethmoid sinuses. Sputum cultures performed during follow-up visits grew *Haemophilus influenza*, methicillin-sensitive *Staphylococcus aureus,* and intermittently, *Pseudomonas aeruginosa*. Cystic fibrosis was excluded by normal sweat test, normal nasal potential difference, and a negative genetic screen for common CFTR mutations among the Ashkenazi-Jewish population. Nasal nitric oxide levels were reduced (mean 34 ppb), a feature of PCD, and an assessment that is rapidly emerging as a first-line test [4], [47], [48]. However, repeat nasal turbinate epithelial biopsies for ultrastructural analyses of the ciliary axoneme by transmission electron microscopy consistently revealed normal ciliary structure (**Figure** **1C**). Sperm motility analysis was offered, but it was declined by the subject.

A search for homozygous regions was performed on the DNA sample from the proband and revealed five homozygous regions longer than 2 Mb (chr2∶209.1-212.7, chr9∶92.1-96.8, chr11∶47.8-54.9, chr12∶40.4-52.5, chr15∶66.3-69.7 Mb). Haplotype analysis using STR markers D9S287, D11S1313, D12S8, D12S368, D15S131 of the parents and the 5 unaffected sibs retained only regions in chr2 and chr12. Within these regions there were 172 protein-coding genes, though only 17 were annotated in the ciliary proteome [5], and only one, CCDC65, was conserved across all organisms with motile cilia and flagella. DNA sequencing of the 8 exons and the flanking introns regions of CCDC65 revealed a single, novel mutation in exon 6, c.876_877delAT (ch12∶47598804-5), which was predicted to result in a frameshift leading to a termination codon at position 293 (**Figure 1D**). The mutation segregated with the disease in an autosomal recessive pattern, with both parents and four healthy siblings heterozygous for the mutation and one sibling was homozygous for the wild-type allele. The mutation was not present in dbSNP135 or in the Exome Variant Server, (NHLBI Exome Sequencing Project, Seattle, WA), and sequencing the coding exons of *CCDC65* did not reveal mutations in 5 unrelated Ashkenazi-Jewish subjects with PCD. The mutation was found in three of 733 anonymous Ashkenazi-Jewish subjects, indicating a carrier rate of 1∶244 in this community.

**Figure 2:**
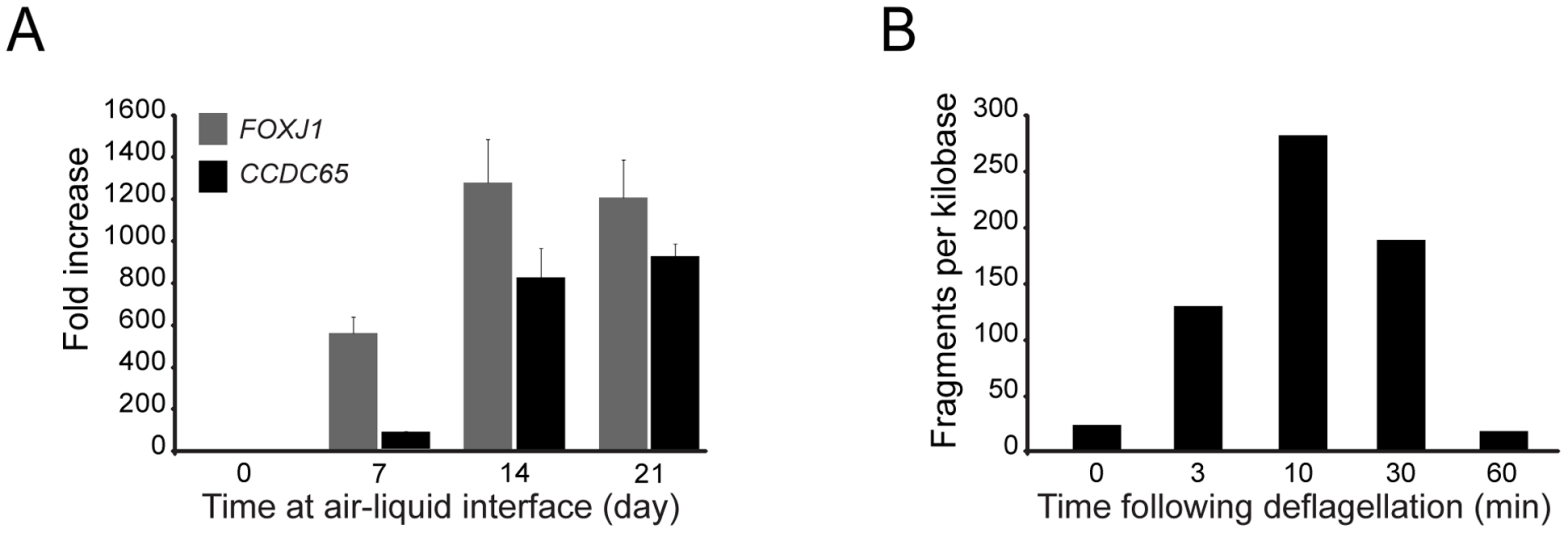
Expression of *CCDC65* and its relationship with *Foxj1* and ciliogenesis. (**A**) *CCDC65* expression, relative to *Foxj1* measured using RT-PCR, increased following the onset of ciliogenesis on air-liquid interface culture days 10 to 14 (p<0.0001; ANOVA). (**B**) *CCDC65* expression determined by RNA sequencing at the indicated times after *C. reinhardtii* deflagellation, which resulted in marked increase in DRC2 expression, the algal *CCDC65* orthologue.

The relationship between *CCDC65* expression and ciliogenesis was examined using primary cultures of hTEC [36]. Cell preparations were allowed to differentiate by initiating an air-liquid interface condition (ALI), and RNA was collected at different time points. *CCDC65* was initially detected during early ciliated cell differentiation (Day 7 post ALI), and coincided with the expression of the master ciliogenesis gene, *FOXJ1* (**Figure 2A**), suggesting that *CCDC65* is associated with ciliogenesis. This relationship was further established by assessing expression of the *CCDC65* orthologue, DRC2 in the biflagellated alga *Chlamydomonas reinhardtii.* RNAseq expression of *CCDC65* increased significantly following deflagellation when compared to pretreatment values, with expression elevated to 6.7-fold at 3 minutes, 13.6-fold at 10 minutes and 7.8-fold at 30 minutes before returning to near baseline (2.1-fold) levels at 60 minutes (**Figure 2B**), consistent with transcriptional up-regulation of flagellar genes during cillogenesis [46].

**Figure 3:**
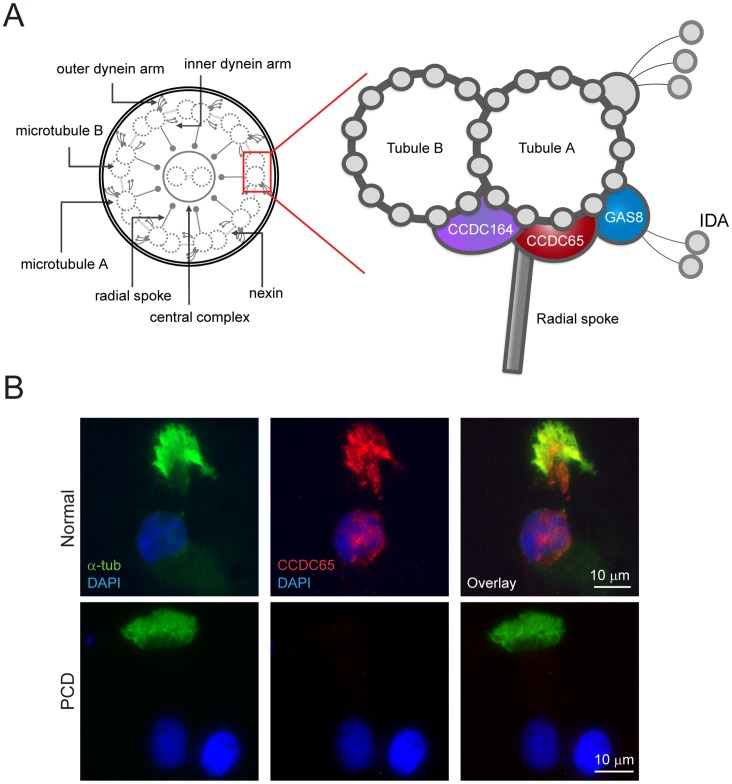
CCDC65 localization in ciliated airway epithelial cells. (**A**) Schematic of a ciliary axoneme cross section, with the N-DRC enlarged (adapted from [30]). (**B**) Photomicrographs of CCDC65 immunofluorescent staining in nasal epithelial cells revealing cytoplasmic and axonemal localization of the protein in normal cells compared to decreased expression in epithelial cells collected from the PCD subject (CCDC65, red; acetylated α-tubulin, green; DAP, blue), Scale bar = 10 µm.

*CCDC65* is the human orthologue of *C. reinhardtii* DRC2, which is a component of the nexin-dynein regulatory complex (**Figure 3A**), a structure important for regulating and orchestrating dynein activity, maintaining outer doublets alignment and limiting the sliding of microtubules during bending [30], [31]. *CCDC65* was previously identified as NYD-SP28, a testis developmental protein that localized to the tail of the sperm [49]. The mutation identified in the affected individual resulted in deletion of nucleotides in *CCDC65* predicted to generate a premature stop codon, resulting in a truncated and likely unstable protein. Motif analysis of the full length-protein CCDC65 showed clusters of phosphorylation, N-glycosylation and N-myristoylation sites, many of which are located at the C’-terminus of the protein. The mutant protein, even if stable would therefore lack important post-translational modification sites [49].

To understand the roles of CCD65 in cilia assembly and function, we evaluated CCDC65 expression and cilia phenotypes in human airway epithelial cells. Nasal epithelial cells obtained from the affected and normal subjects were immunostained for CCDC65 (**Figure 3B**). The protein was detected in the cilia and cytoplasm of airway epithelial cells collected from healthy subjects, but was absent in PCD cells.

**Figure 4:**
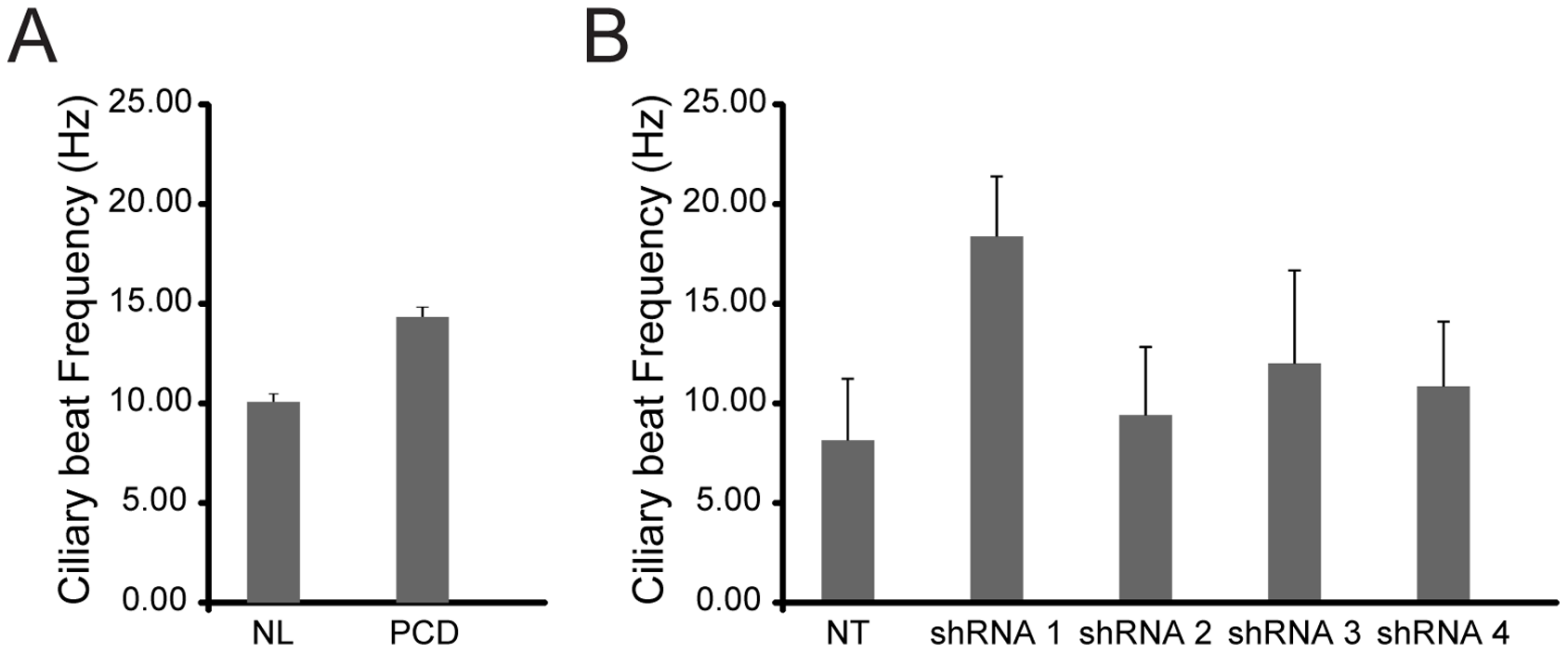
The effect of *CCDC65* shRNA on cilia beat frequency of airway epithelial cells. (**A**) Mean (± standard deviation) of cilia beat frequency in nasal cells from non-PCD (NL) and PCD subject (PCD). (**B**) Mean cilia beat frequency in cells transduced with different targeted *CCDC65* shRNA sequences (shown are the mean ± standard deviation of n = 50 fields, p<0.0001; ANOVA).

The impact of *CCDC65* mutations on cilia function was examined by evaluating the cilia beat patterns using high-speed video imaging. Nasal epithelial cells collected from the PCD subject were assayed as previously described [4], [37], and measured CBF was 14.52±0.07 Hz, compared to 10.89±0.29 Hz from non-PCD subjects (**Figure 4A**). When examining the cilia beat pattern of ciliated cell clusters, the cilia appeared stiff and hyperkinetic, with a limited cilia stroke compared to cilia from non-PCD controls (**video S1 and S2**). To precisely assess ciliary beat patterns and minimize secondary environmental effects on cilia motion [29], *CCDC65* was silenced using RNAi in airway epithelial cells obtained from tracheobronchial tissue of healthy lung transplant donors. Airway epithelial cells were transduced with recombinant lentivirus expressing *CCDC65*-specific shRNA then differentiated at ALI [36]. *CCDC65* mRNA was reproducibly inhibited by different *CCDC65*-specific shRNA sequences when compared to cells transduced with a non-targeted shRNA sequence, as determined using RT-PCR (**Figure S1**). Cilia were present on the apical surface of cells treated with all shRNA sequences, which indicated that *CCDC65* is not required for ciliogenesis. To evaluate the ciliary beat patterns, hTEC were lifted from the culture filters, to form cell clusters that were transferred to glass slides, which permitted cilia motion to be viewed and imaged in the lateral dimension. The CBF measured in cultured hTEC transduced with targeted shRNA sequences was higher than control non-targeted cells (p<0.05, ANOVA with Bonferreoni correction; n = 5 captured fields per Transwell culture, 10 Transwell samples per shRNA sequence) (**Figure 4B**). The variability in CBF observed in the different *CCDC65*-targeted shRNA groups is a reflection of the degree of gene suppression. Moreover, beating cilia appeared dyskinetic, with limited bending at the proximal portion of the cilia, which contributes to a reduced stroke (**video S3, S4, S5, S6**).

**Figure 5:**
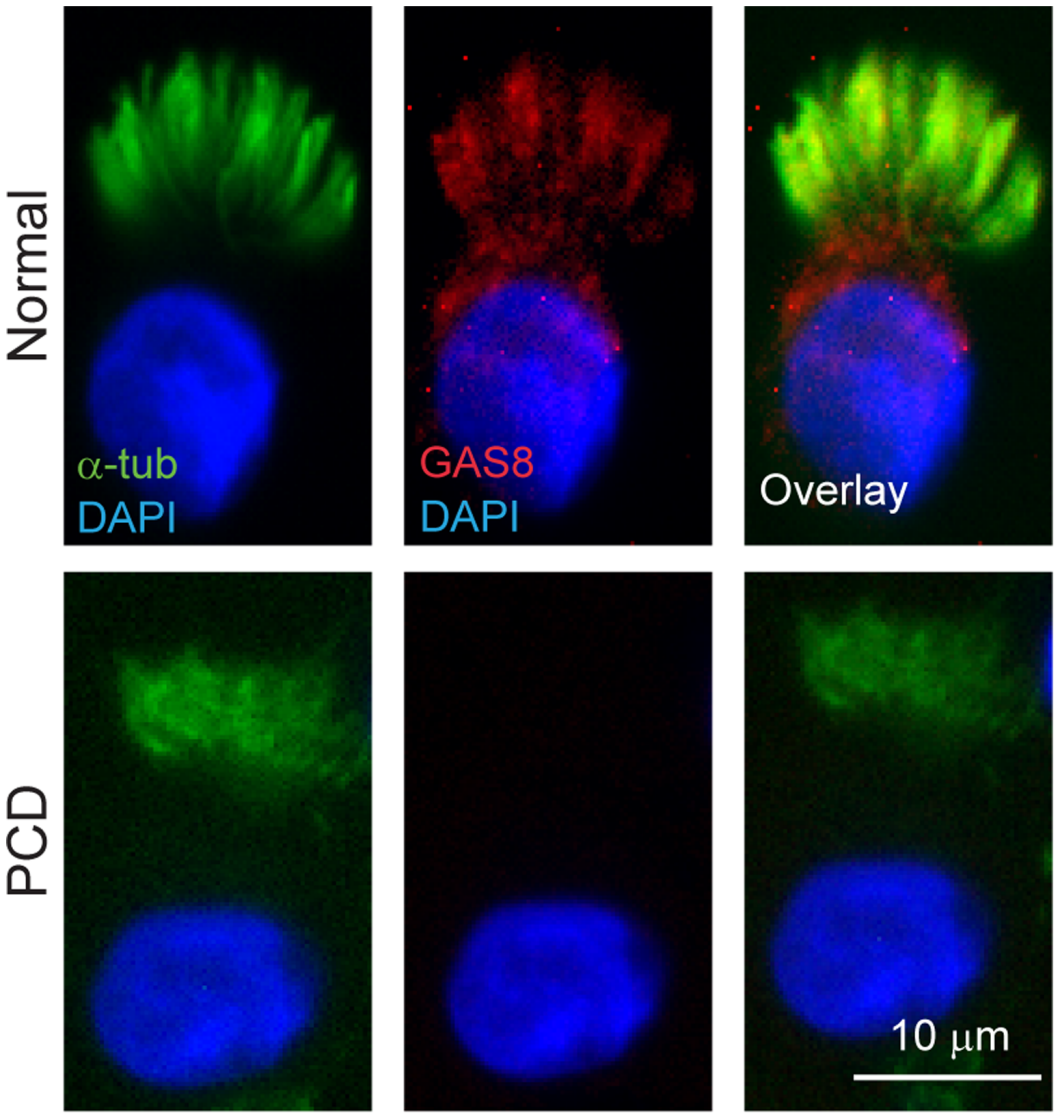
*CCDC65* is involved in nexin-dynein regulatory complex (DRC) assembly. Photomicrographs of normal airway epithelial cells following immunofluorescent staining for GAS8, a component of the N-DRC, which revealed ciliary localization of the protein but markedly reduced levels in nasal epithelial cells from the PCD subject (GAS8, red; acetylated α-tubulin, green; DAPI, blue). Scale bar = 10 µm.

To determine how mutant CCDC65 contributes to altered cilia motion, we evaluated changes in another N-DRC protein. GAS8, also known as DRC4, is a microtubule-binding protein that is an integral part of the N-DRC. Gas8 maintains the alignment between outer doublet microtubules during axonemal bending and is proposed to be positioned in contact with DRC2 in *C. reinhardtii* [31], [50]. Like CCDC65, immunostaining revealed that GAS8 was absent in ciliated nasal epithelial cells isolated from the PCD subject when compared to cells collected from normal volunteers (**Figure 5**). This finding suggests a role of CCDC65 is the assembly and maintenance of the protein components of the N-DRC and thus the regulation of cilia motion.

Changes in the N-DRC are subtle and might be missed or not detected using standard transmission electron microscopy. This is also the case for a PCD-causing mutation in CCDC164 (DRC1), another member of the N-DRC, that results in very minor changes in transmission electron micrographs and could be easily overlooked [28]. Recent studies from large multicenter consortia have shown that roughly one-third of individuals with clinical and diagnostic features consistent with PCD do not have ultrastructural changes, further emphasizing the need for better diagnostic testing [4], [15], [51]. Moreover, given the role of the N-DRC in regulating cilia motility, it is possible that mutations in genes that code for additional members of this complex account for PCD in some cases.

While the number of genotypes causing PCD is expanding, we cannot yet identify specific genotype-phenotype relationships. Of interest, the PCD subject we describe with a mutation in *CCDC65* did not have laterality defects, similar to other reported PCD subjects with mutations in other N-DRC genes [28]. Moreover, based on the medical history obtained from our reported family, we have no evidence of laterality defects associated with this family. However, we cannot exclude the possibility that *CCDC65* mutations can also cause randomization of left-right body asymmetry in humans. Moreover, *CCDC65* was previously localized to the tail of the sperm [49], and based on the observed effects of mutations in *CCDC65* on cilia motility; it is possible that PCD subjects harbouring these mutations have reduced fertility.

In conclusion, we describe a PCD-causing mutation in a novel gene, *CCDC65*, from a subject with classic clinical features and markedly reduced nasal nitric oxide levels, but normal axonemal ultrastructure and subtle impairment of ciliary motility. This contributes to the expanding number of mutations associated with this genetically heterogeneous disease, indicates the important role of the N-DRC in normal airway epithelial cell function, and the importance of developing sensitive and specific screening tests for PCD.

## Supporting Information

Figure S1 ***CCDC65*** **expression in normal human airway epithelial cells following RNAi transduction.** Primary culture human airway epithelial cells were transduced with non-targeted or *CCDC65*-specific shRNA using lentivirus. Following differentiation using air-liquid interface conditions, RNA was isolated and *CCDC65* expression assessed. RT-PCR demonstrated efficient silencing of *CCDC65* expression using different shRNA sequences (shRNA1, shRNA2, shRNA3) compared to non-targeted controls (NT).
(TIF)

Video S1 **Nasal epithelial cells from a subject with PCD at normal speed.** Nasal epithelial cells isolated from the PCD subject with the *CCDC65* mutation at normal speed.
(MP4)

Video S2 **Nasal epithelial cells from a subject with PCD at reduced speed.** Nasal epithelial cells isolated from the PCD subject with the *CCDC65* mutation at reduced speed showing dyskinetic cilia motion.
(MP4)

Video S3 **Non-targeted shRNA transduced cultured hTEC at normal speed.** Non-targeted shRNA-treated hTEC, shown at normal demonstrated normal cilia motion.
(MP4)

Video S4 **Non-targeted shRNA transduced cultured hTEC at reduced speed.** Non-targeted shRNA-treated hTEC, shown at reduced speed demonstrated normal cilia motion.
(MP4)

Video S5 ***CCDC65*** **shRNA transduced hTEC at normal speed.** hTEC transduced with *CCDC65* specific shRNA, shown at normal speed, revealing dyskinetic cilia motion.
(MP4)

Video S6 ***CCDC65*** **shRNA transduced hTEC at reduced speed.** hTEC transduced with *CCDC65* specific shRNA, shown at reduced speed, revealing dyskinetic cilia motion.
(MP4)
