# Supplementary figures and images for: CCDC65 Mutation Causes Primary Ciliary Dyskinesia with Normal Ultrastructure and Hyperkinetic Cilia
Source: PLoS One. 2013 Aug 26;8(8):e72299. doi: 10.1371/journal.pone.0072299 (PMC3753302; doi:10.1371/journal.pone.0072299)

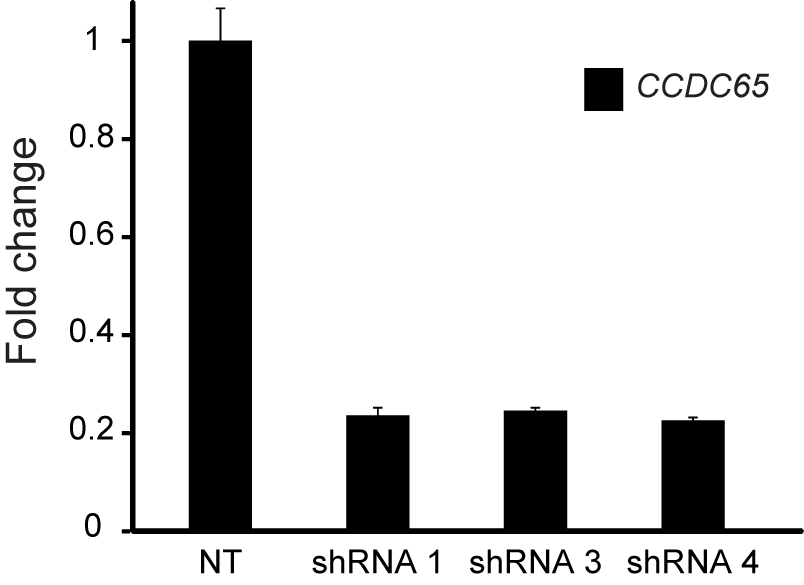

Supplement: Figure S1 — CCDC65 expression in normal human airway epithelial cells following RNAi transduction. Primary culture human airway epithelial cells were transduced with non-targeted or CCDC65-specific shRNA using lentivirus. Following differentiation using air-liquid interface conditions, RNA was isolated and CCDC65 expression assessed. RT-PCR demonstrated efficient silencing of CCDC65 expression using different shRNA sequences (shRNA1, shRNA2, shRNA3) compared to non-targeted controls (NT). (TIF) [file pone.0072299.s001.tif]
